# Scaling Up Physical Activity Promotion Projects on the Community Level for Women in Difficult Life Situations and Older People: BIG-5 and GET-10—A Study Protocol

**DOI:** 10.3389/fpubh.2022.837982

**Published:** 2022-04-14

**Authors:** Maike Till, Karim Abu-Omar, Annika Herbert-Maul, Tobias Fleuren, Anne Kerstin Reimers, Heiko Ziemainz

**Affiliations:** Department of Sport Science and Sport, Friedrich-Alexander Universität Erlangen-Nürnberg (FAU), Erlangen, Germany

**Keywords:** scale-up, socially disadvantaged, physical activity, community-based participatory research, low socioeconomic status, ethnic minorities, vulnerable groups

## Abstract

**Introduction:**

Physical inactivity is a major risk factor for a population's health, especially among socially disadvantaged groups. Many health promotion projects focus on increasing physical activity among their respective target groups. However, because they are mostly developed and implemented under laboratory conditions, they fail when being scaled to real-world settings. The community-based participatory research projects BIG and GESTALT have demonstrated their effectiveness regarding the physical activity promotion in real-world settings by employing a participatory method.

**Material and Analysis:**

Within the context of the BIG-5 and GET-10 projects, these previously implemented and tested participatory projects are scaled to 15 additional settings in Bavaria, Germany. By applying an overarching mixed-methods evaluation framework, the aim is to gain insights into a) the recruitment of communities for scale-up; b) the specific results of the projects according to the RE-AIM framework. In the recruitment of communities, standardized information on the first contact, the consultation process, the person in charge, and previously implemented health promotion projects are collected. A systematic web search will complete information on each community and their health promotion activities. Results will be compared with information on those communities most in need, here according to the deprivation index of communities in Bavaria, Germany. The scale-up process and its results will be measured using semi-structured interviews with project coordinators. A standardized questionnaire will be used with the course's participants. Applying the RE-AIM framework, the collected data will be analyzed deductively.

**Discussion:**

We expect the results to be highly relevant for the effective scale-up of any health promotion project. The study will enhance the understanding of how to reach those communities most in need of health promotion projects and will identify the barriers coordinators face in reaching socially disadvantaged groups.

**Conclusion:**

Although participatory projects are often used as individual projects, little is known about the scaling up of participatory health promotion projects. This cross-cutting evaluation of two projects aims at producing data on the barriers and facilitators for the reach of settings most in need, and those success factors for a durable implementation.

## Introduction

As in many other Western nations, physical inactivity is a major public health issue in Germany (1, 2). Physical inactivity influences population's health in many negative ways, such as being a cause of various non-communicable diseases, including type 2 diabetes, colon cancer, and breast cancer (3, 4). Recent studies have shown that population's physical activity (PA) levels have remained rather unchanged over time (5). In particular socially disadvantaged groups are less likely to meet the recommended level of PA set by the World Health Organization (6, 7).

To target these existing health inequalities, and in particular promote PA, the German Federal Ministry of Health has launched a policy initiative (“IN FORM”) in 2008 (8). This has resulted in numerous projects focusing on PA promotion for different target groups by using a diverse set of individual, setting-based, and also national-level health promotion endeavors (9). While many of these projects have been successful, they have also led to immediate discussion on how to scale “successful” pilot interventions in Germany to increase their public health impact (10, 11). These discussions match the international discourse on that topic (12–14). Scale-up is defined by the World Health Organization (15) as “deliberate efforts to increase the impact of successfully tested health innovations to benefit more people and to foster policy and program development on a lasting basis.” Factors that can lead to the successful scale-up of interventions have been identified by Yamey (16): (1) a simple and technically sound intervention, (2) strong leadership, (3) active engagement of various stakeholders and the target group, and (4) incorporated research. However, it has also been shown that, while scaling-up an intervention, observed effects might diminish, most likely due to adaptation processes (12).

These findings make research on how to scale proven PA interventions, and at the same time maintain their efficacy a relevant research topic. Thus far, only few studies have explored such questions in Germany, particularly for projects rooted in community-based participatory health promotion with the goal of promoting PA.

The two projects titled “*Bewegung als Investition in Gesundheit*” (BIG—interpreted as “Movement as investment in health”) and “*GEhen, Spielen und Tanzen Als Lebenlange Tätigkeit*” (GESTALT—interpreted as “Walking, playing, and dancing as lifelong activities”), described in this study protocol, lend themselves excellently to increase our understanding on the scale-up of such interventions. Both projects have demonstrated to be effective on the individual, policy, and environmental levels, and have succeeded in increasing PA levels among the socially disadvantaged groups of women in difficult life situations and older people with a risk for dementia (17, 18). The GESTALT project increased PA levels outside the class setting for 60% of its participants (17). The BIG project led to increased PA among women, while also having a positive impact on their blood pressure and stress levels (19). The project was able to implement a variety of PA classes according to the needs of the women living in the respective setting (20), hence contributing to their empowerment (21). Additionally, both projects have been, over the years, transferred to other communites. However, this scale-up has to date been rather slow, with an average of one additionally community per year adopting either BIG or GESTALT. Key lessons learned from the previous attempts to scale-up the BIG project have been described (18): This includes, the challenge of creating and maintaining structural support for the project work within community administrations, and having a “champion” for the project in the community. Strong facilitators for success are the continuous involvement of the target group in planning all project activities. For GESTALT, similar observations have been made.

The current study's protocol describes the efforts of the BIG-5 and GET-10 projects to scale up the previously implemented projects BIG and GESTALT to 15 new communities, and thus provide an opportunity to study in depth the facilitators and barriers of scaling-up community-based participatory research projects.

## Aims and Objectives

We aim at scaling up the two community-based participatory research interventions to an additional five (BIG-5) and 10 (GET-10) communities. To the best of our knowledge, this is the first study that includes two separate projects in their evaluation. In doing so, we aim at gaining knowledge on the following key questions, which can be organized into two areas.

### Recruiting Communities for Scale-Up

- How do the communities and rural districts receive information on the two projects?
- Which communities intend to participate in the projects?
- Which barriers do communities face when applying for state-level funding for implementation?

### Results of Project Implementation

- What are the health promotion capacities developed by communities during the project's implementation?
- What are the barriers and facilitators of communities for implementing the projects and for local capacity building?
- Do communities succeed in reaching the target population? If so, to what extent?

By including two projects, namely GET-10 and BIG-5, with a focus on different audiences, we will be able to compare the data regarding the efforts of communities to implement and maintain projects for the respective target groups. Additionally, it increases the chances to produce generalizable knowledge on scale-up and transfer of health promotion interventions.

## Projects and Funding

### BIG-5

The previous project BIG, which forms the foundation for BIG-5, was initiated in 2005. Its aim was to develop and implement exercise health promotion programs among women in difficult life situations. These women include low-income earners, ethnic minority members, the unemployed, those unable to work, and single mothers. BIG has been implemented in 17 communities, and its effects, efficiency, and long-term implementation have been evaluated regularly among the different settings (22). The project created low-barrier PA programs by adopting a participatory research method. By using this method, BIG intended to empower women to make decisions that positively influence their health. Currently, 7 out of the 17 communities were able to offer PA programs (18), with project activities being stopped on average after 4.2 years in 10 communities (18). In the context of BIG-5, our aim is to transfer the project to five additional communities (e.g., municipalities and rural districts) with a special focus on its permanent implementation.

To implement BIG-5, communities receive funding covering a part-time (20 h/week) coordinator position within the communities' administration, and yearly compensation for the voluntary efforts of three peers, to implement the participatory approach, PA classes, and constant advertisement for a period of up to 3.5 years. During funding a total of 18 different PA classes are meant to be implemented in each community. Peers mainly act as social catalysts to help reach out to and involve the target group. Coordinators will be in contact with and receive help from the scientific project team. Table 1 presents a detailed timeline of the BIG-5 project.

**Table 1:** BIG-5 timeline of the project implementation by coordinators.

	**1st year**												**2nd year**												**3rd year**												**4th year**					

**Work-packages (WP)**	**I**			**II**			**III**			**IV**			**I**			**II**			**III**			**IV**			**I**			**II**			**III**			**IV**			**I**			**II**		
	1	2	3	4	5	6	7	8	9	10	11	12	13	14	15	16	17	18	19	20	21	22	23	24	25	26	27	28	29	30	31	32	33	34	35	36	37	38	39	40	41	42
**WP 1: Networking**																																										
Get in contact with potential partners																																										
Kick-off event																																										
Find participating peers																																										
Peer education																																										
**WP2: Analysis**																																										
Analysis of existing community data																																										
Development and execution of a target group need with peers																																										
**WP3: Planning**																																										
Cooperative planning																																										
Maintenance planning of BIG-5																																										
**WP 4: Implementation**																																										
Implementation of PA offers																																										
Training of new intercultural sport trainers																																										
Advertisement of offers																																										
**WP5: Evaluation**																																										
Self-evaluation of capacity building																																										
Participation semi-structured interviews																																										

*The grey shades are indicating the timeline for the several tasks*.

### GET-10

The first GESTALT project was developed and implemented in 2010 and acts as the foundation of GET-10. The project aimed at the prevention of dementia by enabling sedentary older people (above 65 years of age) to develop an active lifestyle. GESTALT has been previously implemented in 5 communities. The project implements an evidence-based 12-session exercise program (Appendix 1) (17). Additionally, trips to other existing exercise programs in the community and integrated health-related counseling are included in the GESTALT project. GET-10 is now transferring the GESTALT project to additional 10 communities that are implementing a total of 4 cycles per community of the pre-developed exercise program over the course of project funding. In addition to the pre-developed exercise programm, GET-10 will use the cooperative planning method (23, 24) to plan the sustainable implementation of the project within the communities from the very beginning. Compared with the BIG-5 project, GET-10 starts with a predeveloped exercise course (a detailed description is provided in Appendix 1). Instructors for the GESTALT program received a specific training course. Table 2 presents a detailed timeline of the GET-10 project.

**Table 2:** GET-10 timeline of project implementation by coordinators.

	**1st year**												**2nd year**												**3rd year**												**4th year**					

**Work-packages (WP)**	**I**			**II**			**III**			**IV**			**I**			**II**			**III**			**IV**			**I**			**II**			**III**			**IV**			**I**			**II**		
	1	2	3	4	5	6	7	8	9	10	11	12	13	14	15	16	17	18	19	20	21	22	23	24	25	26	27	28	29	30	31	32	33	34	35	36	37	38	39	40	41	42
**WP 1: Networking and structures**																																										
Build a network																																										
Education of coordinators (8 workshops)																																										
Peer education																																										
**WP2: Analysis**																																										
Analysis of capacities of PA Promotion																																										
Adjustments for target group																																										
**WP3: Planning**																																										
Cooperative planning																																										
Maintenance planning of GET-10																																										
**WP 4: Implementation**																																										
GET-10 offer																																										
New offers																																										
Training of new trainers																																										
Advertisement of offers																																										
**WP5: Evaluation**																																										
Self-evaluation of capacity building																																										
Participation semi-structured interviews																																										

*The grey shades are indicating the timeline for the several tasks*.

## Methods and Analysis

The projects receive funding from the Federal Centre for Health Education. Communities located in Bavaria, Germany, with over 5,000 inhabitants can apply for funding for one of the projects by contacting the scientific coordinators. Using a low-threshold application scheme, communities have to send an expression of interest signed by the local major to the funding agency. Based on the expression of interest and some other formal criteria, communities receive funding.

During the project work, the 15 communities function as so-called “real-world laboratories” that “contribute to capacity development, new scientific insights and societal learning, building on iterations of experimentation and reflection” (25). In this process, real-world laboratories aim at meeting four characteristics: (1) follow transformative science approaches (e.g., testing out solutions to reduce health inequalities), (2) establish the mechanisms of coproduction between science and health promotion practice, (3) follow transdisciplinary approaches (i.e., scientific, policy, and practice) and to enable the target population to work together in finding possibilities of intervention implementation and sustainability of the project, and (4) plan for a durable scaling-up of implementation (26).

Communities will implement a predeveloped project plan for one of the two projects, as visualized in Tables 1, 2. During implementation, they will receive guidance and scientific support. Both projects are similar as both target disadvantaged population groups and adopt a participatory approach that enables communities to establish and sustain target group-specific interventions for PA. Using the participatory method of cooperative planning (23, 24), the projects aim to include members of the target group, researchers, community administrative staff, and practitioners (e.g., staff from local sport clubs or other health departments) in the planning and implementation of interventions for promoting PA among the target group. Coordinators will receive a manual on how to conduct the cooperative planning method (27), including a detailed description of all the steps. Based on the participatory nature of the projects, some actions taken may vary between the communities.

Coordinators also receive guidance and attend biannual workshops to share experiences among themselves. The workshops will be organized by the scientific team and cover topics such as (a) the cooperative planning method and coproduction of knowledge, (b) networking and capacity building, and (c) empowerment of the target group. Additional topics will be raised by coordinators. Because both projects follow the same participatory methodology, they work closely together and are connected during workshops and knowledge-exchange meetings.

Both projects have been approved by the ethics committee of FAU, Germany (520_20Bc). All participants who are involved will be informed about the voluntary nature of participation in any scientific data collection and their right to withdraw their consent at any time without having to provide a reason.

To answer the research questions in the three areas mentioned above, a convergent parallel mixed methods approach (28) will be applied. We will use concurrent implementation of quantitative and qualitative strands which will be compared and related during the analysis to consolidate our findings.

### Recruiting Communities for Scale-Up

To evaluate the recruitment process of the communities, all contact between the researchers and the interested community members will be systematically documented. Interested community members will engage in one-on-one phone calls with researchers. During the call, the researchers will ask standardized questions to gather information on (a) how the communities learned about the projects (e.g., via newsletters, social media posts, or through colleagues), (b) who contacted the researchers (e.g., mayor, health department, external partner, etc.), (c) why they were interested in the project, and d) if they had previous experience or carried out similar projects in the past.

Additionally, data will be collected on all the interactions with the communities until they have made a final decision to take part. This will include (a) the time it took, (b) the number of contacts, and (c) the factors that the communities mentioned as influencing their decision.

A systematic web and database search and qualitative content analysis, here according to McMillan et al. (29), will complement the systematic documentation by identifying each community's (1) population size, (2) community deprivation index developed by Kroll (30), (3) previously implemented health promotion programs, and (4) the percentage of target population among inhabitants and other potentially relevant factors (e.g., party alignment in city councils). To generate additional data on the communities most in need, the community deprivation index will be utilized (30).

The collected data will be analyzed using qualitative content analysis (31) to make comparisons among the following three groups: (a) participating communities, (b) interested communities that did not apply for funding, and (c) communities most in need according to the community deprivation index that match the funding criteria (located in Bavaria with inhabitants totaling over 5,000).

The analysis will identify the time period needed by communities to reach a decision, barriers and facilitators that led to the decision, number of already existing health-promotion projects in the communities, and whether socioeconomically deprived communities are being reached by the funding scheme.

### Results of Project's Implementation

To answer the research questions regarding the effectiveness of the projects, we utilize the RE-AIM framework and its five dimensions: “reach,” “efficacy,” “adoption,” “implementation,” and “maintenance” (32). Using the RE-AIM framework allows us to address our research questions in regard to the ability of communities to generate public health impact by implementing the two exercise interventions which are targeted at vulnerable groups. Additionally, as mentioned by Kwan et al. (33) it “provides structure to systematically evaluate health program impact.”

We will conduct annual semi-structured interviews with the project's coordinators of the 15 communities (*n* = 45 interviews), as well as annual semi-structured focus groups with peers (*n* = 30 interviews) in each setting. Additionally, quantitative data will be collected, including an annual evaluation of capacity building using a standardized questionnaire filled out by the projects' coordinators, all attendance lists of the BIG-5 and GET-10 exercise courses, including number of people on the waiting lists, and data on satisfaction using a standardized short feedback questionnaire filled out by the course participants. Table 3 describes all study evaluation methods, participants, and study parameters.

**Table 3:** Evaluation methods.

		**Participants**	**Time of measurement**	**Topics of interest**	**Total**	**RE-AIM indicator**
Recruiting communities for scale-up	Semi-structured telephone interviews	All interested communities		- Who contacted the researcher? - How did they receive information? - Is there a specific reason why they want to implement the project? - Do they have previous or similar health promotion projects?		-
	Timeline documentation	All interested communities	From first contact until decision making or project implementation	- How long do communities take until making a decision? - Do we reach communities that are the most in need?		-
	Web-based search	Participating communities + interested communities + 10 most deprived Bavarian communities		- How active are communities regarding health promotion? - Do we reach communities that are the most in need? - Percentage of the target population in the community		-
Results of projects' implementation	Semi-structured interviews	Community coordinators (*n* = 15)	Annually	- Barriers and facilitators in reaching the target population - Adaptations made to the cooperative planning method - Capacity-building areas: (1) participation, (2) local leadership, (3) available resources, (4) networking and cooperation, (5) health care - Efforts made for the durable implementation	*n* = 45	Effectiveness, adoption, implementation, maintenance
	Semi-structured focus group	Peers (*n* = 15)	Annually starting from year 2	- Barriers and facilitators during work as peers - Satisfaction with the courses - Empowerment of peers Capacity-building areas: (1) participation, (2) local leadership, (3) available resources, (4) networking and cooperation, and (5) health care	*n* = 30	Effectiveness, adoption, implementation
	Attendance lists		Every course	- Reach of target population		Reach, effectiveness
	Waiting lists		Every course	- Reach of target population		Reach, effectiveness
	Satisfaction questionnaire	Participants of courses	After each course	- Satisfaction with the course incl. time, length, difficulty, trainer, and instructions		Effectiveness
	Capacity-building questionnaire	Community coordinators (*n* = 15)	Annually	-Capacity-building areas: (1) participation, (2) local leadership, (3) available resources, (4) networking and cooperation, (5) health care	*n* = 45	Adoption, implementation, maintenance

#### Reach

During the annual interviews, coordinators will be asked about the efforts made to reach the specific target group, as well as the barriers and facilitators in reaching participants. Because peers of the target group act as social catalysts in recruiting participants and in advertising, they will be asked to report on their experiences, including the barriers and facilitators in reaching the target population. Additionally, attendance lists of exercise courses in each community will be gathered, including the number of participants on potential waiting lists. These data will be used to analyze how offers reach the target group. We will look specifically at the differences in reach between GET-10 and BIG-5.

#### Efficacy

Efficacy will be assessed in the GET-10 project by questionnaire to assess the PA levels of the participants in the classes. Efficacy in the BIG-5 project will be assessed by the attendance lists by looking into the demographics of course participants and compare them to overall demographics of the community.

#### Adoption

The information gathered throughout the recruitment process of the 15 communities will be utilized to gain insights into which communities take part in the projects and how they differ from those not taking part.

#### Implementation and Maintenance

Because both BIG-5 and GET-10 apply the participatory method of cooperative planning and aim at a durable implementation, the RE-AIM dimensions of *implementation* and *maintenance* can also be considered the effectiveness of the implementation strategies. Annually conducted semi-structured interviews with coordinators will collect data on the following topics:

- Adaptations made to the cooperative planning method by communities.
- Efforts made for implementation over time.

Semi-structured interviews (*n* = 45) and focus group interviews (*n* = 30) will be audio-recorded, transcribed verbatim, and anonymized. Data will be deductively coded using Mayring's qualitative content analysis (31). Codes will focus on the barriers and facilitators regarding the RE-AIM dimensions (32). Additionally, we will analyze the results regarding (1) the population size of the community, (2) the community's deprivation index developed by Kroll (30), and (3) county or city implementation.

As theoretical guidance we will make use of Trojan and Nickel's framework of capacity building (34). This framework is based on Laverack and Labone (35, 36) initial work and covers a total of five dimensions of capacity building: (1) participation, (2) local leadership, (3) available resources, (4) networking and cooperation, and (5) health care.

Additionally, we will use the Cosolidated Framework for Implementation Research (CFIR) (37). This framework allows a detailed mapping of all factors that have positive or negative impact on the implementation of the intervention in the communities. The framework will serve as a guidance for the deductive analysis of the qualitative data.

Based on the capacity building dimensions and stimulated by a study by Nickel et al. (38), we have developed a capacity-building questionnaire that fits the project's goals, target population, and participatory methodology. The questionnaire will be tested as part of the project work using the “think-aloud” method (39). A preliminary version can be found in the appendix (Appendix 2). The participants of the “think-aloud” study will be coordinators from five former BIG and GESTALT communities (*n* = 5).

A survey will be sent annually to the projects' coordinators (*n* = 45). Survey data will be analyzed using spider graphs for easy visualization of the data for each community, as suggested by Trojan and Nickel (34). Designed as self-monitoring tools for communities, a pre-defined analysis Excel sheet that automatically creates spider graphs, including the different capacity areas (e.g., participation, local leadership, available resources, networking, and cooperation, and health care) and their subcategories, will be shared with the communities (Appendix 3). This will serve as part of the quantitative string in our convergent parallel mixed methods approach.

Survey data of the different communities will be compared with the demographic data of the communities to assess how communities from different deprivation statuses are able to built capcities for PA-related health promotion. Further, data will be compared regarding differences among communities that implement measures for the elderly (GET-10) and women in difficult life situations (BIG-5).

To receive further in-depth information on the capacity areas defined by Nickel et al. (38), semi-structured interviews will be conducted annually with the project coordinators (*n* = 45). Therefore, questions will ask for (1) the participation of local stakeholders and the target population during the cooperative planning sessions, (2) the contributions of local leaders (e.g., major, sport clubs, etc.), (3) available resources (e.g., rooms, financials, trainers, etc.), (4) networking and cooperation (e.g., efforts made to inform about the projects and find additional partners), and (5) program offers implemented in the community.

To receive information on the effects regarding the empowerment of peers, the latter (three peers per setting) will be asked to participate in annual focus group interviews (*n* = 30). Questions will focus on the efforts made to recruit participants, their satisfaction with the program, and their personal opinion on the capacity-building dimension (as mentioned above).

By analyzing using both qualitative and quantitative methods, we expect to uncover insights that will help future community-based participatory projects during their implementation process.

## Discussion

To date, evidence on the scale-up of PA promotion projects in Germany is scarce. Studying the scale-up of two projects targeting vulnerable population groups has the potential to increase our understanding of how to generate public health impacts.

These two projects can yield valuable information on the barriers and facilitators faced by communities when adopting such interventions. Additionally, the focus of the interventions on vulnerable groups (women in difficult life situations and older people) featuring high levels of physical inactivity makes the projects valuable from a public health and health equity perspective (6). At the same time, the projects demonstate the challenges faced by researchers and health practitioners when attempting to scale-up and sustain health promotion interventions. For one, this pertains to the identification of adequate methods and theoretical frameworks in order to study processes of scale-up. But also, the challenge of striving a balance between “fidelity” (a given intervention is implemented as intended) and “adaptation” (making intentional changes to an intervention to achieve a better fit with the context) and how this might impact project's effectiveness. Lastly, the challenge of scaling and sustaining evidence-based health promotion interventions across a diverse set of communities and target groups. The projects will enhance this process by creating a coproduction process that includes project officers from 15 communities and researchers.

## Strengths and Limitations

The limitations of the present study include the following:

1) Measurements regarding the reach of the population group and effectiveness of the interventions will use a mixed methods approach and mainly rely on qualitative interviews and surveys. A more robust study design (e.g., randomized controlled trial) is not in place, and as such, we will provide only limited information on how effective the two interventions are in generating a public health impact.
2) The study duration is 3.5 years. Thus, the projects will not yield results regarding the long-term impacts of the interventions, in particular on the question of whether they are being maintained in the different communities.
3) Because of the COVID-19 pandemic, we expect a delay in the implementation of projects, which may influence the outcomes of our scale-up process.

Nevertheless, our study also has some strengths:

1) We attract a diverse set of communities, ranging from larger cities (more than 100,000 inhabitants) to rural counties with around 5,000 to 10,000 inhabitants. As such, the communities will differ greatly in their potential to implement the two interventions, which will give greater insights into the diversity of communitie's implementation capacities.
2) Both projects can draw on empirical evidence gathered over 17 (BIG) and 12 (GESTALT) years, respectively. This extensive experience can be used for the successful implementation of the two project's successful implementation in 15 new settings.
3) By using a mixed methods approach, we can gain not only insights from a quantitative perspective, but also in-depth qualitative information on all the factors related to the implementation of the interventions in the communities.
4) The sample size of 15 communities is quite generous for studying factors that would allow for further scaling of both projects.
5) The projects allow us to gather information not only on communities who participate, but also on those who voice some interest but then decide against joining the funding scheme. This provides a unique opportunity to identify the barriers that inhibit the scaling of the two interventions.

## Conclusion

Scale-up and sustainment of community-based participatory research projects is crucial to reduce health inequalities. The two interventions have already been evaluated and demonstrated effectiveness. The current study can provide additional evidence on the barriers and facilitators of scale-up and sustainability, capacity building within a community setting through health promotion projects, and the project's effects on the target populations. Through a cross-sectional examination of the recruitment process of communities, the current study will be the first to provide evidence on how to reach communities to engage in such projects and the barriers and facilitators for implementation they may face.

## Author Contributions

AH-M, TF, KA-O, HZ, and AR contributed to conception and design of the study. MT wrote the first draft of the manuscript. All authors contributed to manuscript revision, read, and approved the submitted version.

## Funding

This study received funding from the German Federal Center for Health Education (grant number BIG-5: Z2/95022001; grant number GET-10: Z2/95022002). The funder was not involved in the study design, and the writing of this article or the decision to submit it for publication.

## Conflict of Interest

The authors declare that the research was conducted in the absence of any commercial or financial relationships that could be construed as a potential conflict of interest.

## Publisher's Note

All claims expressed in this article are solely those of the authors and do not necessarily represent those of their affiliated organizations, or those of the publisher, the editors and the reviewers. Any product that may be evaluated in this article, or claim that may be made by its manufacturer, is not guaranteed or endorsed by the publisher.
