# Supplementary material for: Scaling Up Physical Activity Promotion Projects on the Community Level for Women in Difficult Life Situations and Older People: BIG-5 and GET-10—A Study Protocol
Source: Front Public Health. 2022 Apr 14;10:837982. doi: 10.3389/fpubh.2022.837982 (PMC9046678; doi:10.3389/fpubh.2022.837982)
Supplement: Supplementary file 2 [file Data_Sheet_1.PDF]

Appendix 1: Overview of the GESTALT Intervention

Session	Weekly structure	Meeting theme	Coaching/Theory	Main part
1	Step 1: Introduction through Movement & collecting first experience	Get to know each other through movement and Games	Goals and information of GESTALT	Endurance & Strength (Games and exercises with balloons)
2		Learning about the material to the music	Advantages of movement as prevention of dementia & introduction of BORG-scale	Endurance & Coordination (Square Dance)
3		Gathering experience of the material in the parcourse	Rep of the preventive components & reasons of participation	Strength & Balance (Landscape parcourse)
4	Step 2: Preparing the transfer to daily life	Playing together I + Ideas of activities to perform at home	Training of goal setting & Introduction of the participant information	Coordination & Endurance (Ball over the rope)
5		Moving together I + Ideas of activities to perform at home	Consultation about reaching the goals, function of memory & Tips for daily life	Endurance & Strength (Basic steps Aerobic)
6		Walking together I + Ideas of activities to perform at home	Introduction to barrier management & rep. Goal setting	Endurance & Strength („Who am I" & Word pairs with strengthening)
7	Step 3: Transfer to daily life	Playing together II<br>Group excursion to the topic „playing"	Recap and preview of the course & barrier plan I	Strength endurance (exercise and games with swing cloth)
8		Dancing together I<br>Group excursion to the topic „dancing"	barrier plan II & reward	Endurance & coordination (Line Dance)
9		Walking together II<br>Group excursion to the topic „walking"	Advantages: „Movement"	Endurance (Walking according to symbols)

Session	Weekly structure	Meeting theme	Coaching/Theory	Main part
10	Step 4: Sustainability and Self-determination	Playing together III<br>Visiting a trial lesson independently	Independent information about offers & suitability of activities	Coordination & Strength (Games and exercises with balls, sticks and towels)
11		Moving together II<br>Visiting a trial lesson independently	Reflexion on trial lessons & social support	Endurance & Coordination (Square Dance)
12		Walking together III & finalization<br>Visiting a trial lesson independently	Reflexion on the „success“ in sports	Endurance (Robot game & walking with equipment)

Areas: Walking Playing Dancing Coaching Additional offers

Requirement profile: performance capability impression and expression
