# Supplementary material for: Scaling Up Physical Activity Promotion Projects on the Community Level for Women in Difficult Life Situations and Older People: BIG-5 and GET-10—A Study Protocol
Source: Front Public Health. 2022 Apr 14;10:837982. doi: 10.3389/fpubh.2022.837982 (PMC9046678; doi:10.3389/fpubh.2022.837982)
Supplement: Supplementary file 3 [file Data_Sheet_2.PDF]

Kapazitätsentwicklung in der Kommune

Ziel dieser Befragung ist es, Strukturen und Kapazitäten für mehr Prävention und Gesundheitsförderung in Ihrer Kommune zu messen und beschreiben zu können. Die Ergebnisse dienen in erster Linie einer fundierten Dokumentation des Ist-Zustandes und der Veränderungen in Ihrer Kommune rund um das BIG-5 bzw. Get-10 Projekt.

Die Selbstevaluation umfasst folgende Themen:

- **Beteiligung** (wesentliche Elemente, Eigeninitiative, Beteiligung),
- **verantwortliche lokale Führung** (Ziele, inhaltlicher und organisatorischer Ablauf, Steuerungskompetenzen),
- **vorhandene Ressourcen** (Rahmenbedingungen, Ressourcen),
- **Vernetzung und Kooperation** (lokale und überlokale Vernetzung und Kooperation, Öffentlichkeitsarbeit),
- **Gesundheitsversorgung** (Bereitstellung von Angeboten, Überwindung von Zugangsbarrieren, Nachhaltigkeit).

Bei den Fragen bitten wir Sie, die **Situation der letzten 12 Monate** in Ihrer Kommune einzuschätzen. Überlegen Sie bitte, welche der vorgegebenen Antwortmöglichkeiten am besten zutrifft und kreuzen Sie das entsprechende Kästchen an.

Uns ist klar, dass es sich dabei um ihre eigene Einschätzungen handelt.

Wenn Sie eine Frage nicht beantworten können, bitten wir Sie die Kategorie "kann ich nicht beurteilen" anzukreuzen.

Vielen Dank für Ihre Teilnahme!

© 2022 / Department für Sportwissenschaft und Sport, Friedrich-Alexander-Universität Erlangen-Nürnberg

BETEILIGUNG

Bitte geben Sie an, inwieweit die folgenden Kriterien **in den letzten 12 Monaten** in Ihrem BIG- oder GESTALT-Projekt erfüllt wurden.

	Stimme gar nicht zu 1	Stimme weniger zu 2	Stimme teilweise zu 3	Stimme weitest-gehend zu 4	Stimme voll zu 5	kann ich nicht beurteilen
**Wesentliche Elemente des Projekts**						
1. Durch das Projekt werden in unserer Kommune Strukturen für die langfristige Gesundheitsförderung geschaffen bzw. gestärkt.	☐	☐	☐	☐	☐	☐
2. Die Bedürfnisse der Zielgruppe stehen bei allen Aktivitäten im Rahmen des Projekts im Fokus.	☐	☐	☐	☐	☐	☐
3. Es finden ausreichend Planungsgruppen statt, bei denen die Angebote geplant werden	☐	☐	☐	☐	☐	☐
4. Die Moderation trägt dazu bei, dass sich bei den Planungstreffen alle Teilnehmenden gleichermaßen einbringen können.	☐	☐	☐	☐	☐	☐
5. Die Beteiligung der Zielgruppe an der Planung und Umsetzung des Projekts wird durch wirksame Aktivierungstechniken gefördert. *(z.B. durch Informationsarbeit und Multiplikator*innen).*	☐	☐	☐	☐	☐	☐
6. In der Planung des Projekts wird Engagement und Mitwirkung anerkannt und belohnt	☐	☐	☐	☐	☐	☐
7. Personen aus den zuständigen Ämtern und Einrichtungen setzen sich für das Projekt ein.	☐	☐	☐	☐	☐	☐
8. Es gibt ein Netzwerk von Multiplikator*innen, die Projekt bei der Zielgruppe bewerben.	☐	☐	☐	☐	☐	☐

© 2022 / Department für Sportwissenschaft und Sport, Friedrich-Alexander-Universität Erlangen-Nürnberg

	Stimme gar nicht zu 1	Stimme weniger zu 2	Stimme teilweise zu 3	Stimme weitest-gehend zu 4	Stimme voll zu 5	kann ich nicht beurteilen
**Eigeninitiative**						
9. Ich, als Koordinator*in, ergreife selbst Initiative zur Lösung der von mir im Setting wahrgenommenen Probleme.	☐	☐	☐	☐	☐	☐
10. Ich übernehme zunehmend größere Verantwortung für das Projekt.	☐	☐	☐	☐	☐	☐
11. Ich bringe meine Bedürfnisse und Anliegen als Koordinator*in in der Planungsgruppe ein.	☐	☐	☐	☐	☐	☐
**Beteiligung der Zielgruppe**						
12. Die Zielgruppe wirkt aktiv an der Planung und Umsetzung der Angebote mit.	☐	☐	☐	☐	☐	☐

© 2022 / Department für Sportwissenschaft und Sport, Friedrich-Alexander-Universität Erlangen-Nürnberg

VERANTWORTLICHE LOKALE FÜHRUNG

Bitte geben Sie an, inwieweit die folgenden Kriterien **in den letzten 12 Monaten** in Ihrem BIG- oder GESTALT-Projekt erfüllt wurden.

		Stimme gar nicht zu 1	Stimme weniger zu 2	Stimme teilweise zu 3	Stimme weitest-gehend zu 4	Stimme voll zu 5	kann ich nicht beurteilen
**Planung des inhaltlichen und organisatorischen Ablaufs**							
13.	Ich verwende das Manual zur Umsetzung des Projekts	☐	☐	☐	☐	☐	☐
14.	Die gemeinsame Planungsgruppe ist die zentrale Methode zur Umsetzung der Projektziele.	☐	☐	☐	☐	☐	☐
15.	In der Planungsgruppe wurden übergeordnete Ziele definiert. *(Beispiel: Durch niederschwellige Angebote wird der Zugang zu Bewegung erleichtert.)*	☐	☐	☐	☐	☐	☐
16.	In der Planungsgruppe werden konkrete Handlungsziele und Maßnahmen definiert. *(Beispiel: Bis [Datum] werden [Anzahl] niederschwellige Bewegungskurse in [Anzahl] Stadtteilen angeboten.)*	☐	☐	☐	☐	☐	☐
17.	In der Planungsgruppe wurde vereinbart bis wann die konkreten Ziele erreicht werden sollen.	☐	☐	☐	☐	☐	☐
18.	In der Planungsgruppe wurde geklärt, wer für die Umsetzung der Maßnahmen zuständig ist.	☐	☐	☐	☐	☐	☐
**Steuerungskompetenzen der lokalen Führung**							
19.	Das Projekt ist in der Kommune an der richtigen Stelle angesiedelt. *(z.B. Sport- oder Gesundheitsamt, Integrationsbeauftragte/r, GR+).*	☐	☐	☐	☐	☐	☐
20.	Die lokale Koordination schafft es Veränderungsprozesse in der Kommune voranzubringen.	☐	☐	☐	☐	☐	☐
21.	Die Inhalte der Planungsgruppe werden protokolliert.	☐	☐	☐	☐	☐	☐
22.	Es gibt einen jährlichen Bericht über die Aktivitäten des Projekts	☐	☐	☐	☐	☐	☐

© 2022 / Department für Sportwissenschaft und Sport, Friedrich-Alexander-Universität Erlangen-Nürnberg

		Stimme gar nicht zu<br>1	Stimme weniger zu<br>2	Stimme teilweise zu<br>3	Stimme weitest-gehend zu<br>4	Stimme voll zu<br>5	kann ich nicht beurteilen
23.	Definierte Ziele und angewandte Methoden werden gemeinsam in regelmäßigen Abständen reflektiert.	☐	☐	☐	☐	☐	☐
24.	Die Inhalte und Ergebnisse der Planungsgruppe werden innerhalb der Kommune weitergeben und kommuniziert.	☐	☐	☐	☐	☐	☐
25.	Die Erreichung der Ziele wird in regelmäßigen Abständen überprüft.	☐	☐	☐	☐	☐	☐

© 2022 / Department für Sportwissenschaft und Sport, Friedrich-Alexander-Universität Erlangen-Nürnberg

VORHANDENE RESSOURCEN

Bitte geben Sie an, inwieweit die folgenden Kriterien **in den letzten 12 Monaten** in Ihrem BIG-Projekt oder GESTALT-Projekt erfüllt wurden.

	Stimme gar nicht zu 1	Stimme weniger zu 2	Stimme teilweise zu 3	Stimme weitest-gehend zu 4	Stimme voll zu 5	kann ich nicht beurteilen
**Rahmenbedingungen**						
26. In unserer Kommune gibt es genügend Informationen und Analysen zu gesundheitlichen und sozialen Ungleichheiten.	☐	☐	☐	☐	☐	☐
27. In unserer Kommune gibt es einen Bedarf an gesundheitsförderlichen Maßnahmen für die Zielgruppe.	☐	☐	☐	☐	☐	☐
28. In unserer Kommune gibt es eine starke politische Unterstützung für das Projekt.	☐	☐	☐	☐	☐	☐
**Ressourcen**						
29. Es gibt ausreichend Räume zur Umsetzung der Maßnahmen.	☐	☐	☐	☐	☐	☐
30. Es gibt ausreichend Sport-Materialien zur Umsetzung von Bewegungskursen in der Kommune.	☐	☐	☐	☐	☐	☐
31. Es gibt ausreichend Personal für die Koordination des Projekts.	☐	☐	☐	☐	☐	☐
32. Es gibt ausreichend finanzielle Mittel für die Umsetzung von Bewegungsmaßnahmen.	☐	☐	☐	☐	☐	☐
33. Es gibt ausreichend Übungsleiterinnen für Bewegungsmaßnahmen	☐	☐	☐	☐	☐	☐
34. Der Aufwand für das Projekt steht in einem guten Verhältnis zu den Ergebnissen, die das Projekt erzielt.	☐	☐	☐	☐	☐	☐

© 2022 / Department für Sportwissenschaft und Sport, Friedrich-Alexander-Universität Erlangen-Nürnberg

VERNETZUNG UND KOOPERATION

Bitte geben Sie an, inwieweit die folgenden Kriterien **in den letzten 12 Monaten** in Ihrem BIG- oder GESTALT-Projekt erfüllt wurden.

	Stimme gar nicht zu 1	Stimme weniger zu 2	Stimme teilweise zu 3	Stimme weitest-gehend zu 4	Stimme voll zu 5	kann ich nicht beurteilen
**Lokale Vernetzung und Kooperation**						
35. Alle Beteiligten sind offen für den Austausch.	☐	☐	☐	☐	☐	☐
36. Involvierte Personen und/oder Institutionen bilden innerhalb der Kommune Bündnisse und Partnerschaften.	☐	☐	☐	☐	☐	☐
37. Die Vernetzungen und Kooperationen der lokalen Personen und/oder Institutionen untereinander sind ausgezeichnet.	☐	☐	☐	☐	☐	☐
38. Die lokalen Partner*innen besitzen die notwendigen Kooperationskompetenzen. *(Beispiel: Kommunikations- und Konfliktlösefähigkeiten)*	☐	☐	☐	☐	☐	☐
39. Die lokalen Partner*innen nutzen vorhandene Netzwerke, um Probleme zu bewältigen und Ressourcen für das Projekt zu erschließen.	☐	☐	☐	☐	☐	☐
40. Die lokalen Partner*innen arbeiten effektiv und zielgerichtet zusammen.	☐	☐	☐	☐	☐	☐
**Überlokale Vernetzung und Kooperation**						
41. Es findet ein überregionaler Austausch mit Projektkoordinator*innen von anderen Standorten statt.	☐	☐	☐	☐	☐	☐
**Öffentlichkeitsarbeit**						
42. Mit der öffentlichen Wahrnehmung des Projekts in unserer Kommune bin ich zufrieden.	☐	☐	☐	☐	☐	☐

© 2022 / Department für Sportwissenschaft und Sport, Friedrich-Alexander-Universität Erlangen-Nürnberg

GESUNDHEITSVERSORGUNG

Bitte geben Sie an, inwieweit die folgenden Kriterien **in den letzten 12 Monaten** in Ihrem BIG- oder GESTALT-Projekt erfüllt wurden

		Stimme gar nicht zu 1	Stimme weniger zu 2	Stimme teilweise zu 3	Stimme weitest-gehend zu 4	Stimme voll zu 5	kann ich nicht beurteilen
**Bereitstellung von BIG oder GESTALT Angeboten**							
43.	In unserer Kommune gibt es mehrere Angebote zur Förderung der Gesundheit der Zielgruppe.	☐	☐	☐	☐	☐	☐
44.	Die Angebote in unserer Kommune sind gut besucht.	☐	☐	☐	☐	☐	☐
45.	Wenn ein Kurs erstmal gestartet ist, bleibt er im Angebot.	☐	☐	☐	☐	☐	☐
46.	Die an teilnehmenden Personen bleiben dabei.	☐	☐	☐	☐	☐	☐
47.	Durch das Projekt werden benachteiligte Zielgruppen erreicht.	☐	☐	☐	☐	☐	☐
48.	Alle Personen der Zielgruppe, die Interesse haben, können teilnehmen.	☐	☐	☐	☐	☐	☐
**Überwindung von Zugangsbarrieren**							
49.	Unsere Angebote sind kostengünstig.	☐	☐	☐	☐	☐	☐
50.	Unsere Angebote sind wohnortnah.	☐	☐	☐	☐	☐	☐
51	Unsere Angebote sind ohne Vertragsbindung.	☐	☐	☐	☐	☐	☐
52.	Unsere Angebote sind bar bezahlbar.	☐	☐	☐	☐	☐	☐
53.	Unsere Angebote entsprechen den Wünschen der Teilnehmer*innen.	☐	☐	☐	☐	☐	☐
54.	Die Multiplikator*innen und Peers werben in unterschiedlichen Lebensbereichen für das Projekt *(Beispiel: Moschee, Kita, Gemeindefest)*	☐	☐	☐	☐	☐	☐

© 2022 / Department für Sportwissenschaft und Sport, Friedrich-Alexander-Universität Erlangen-Nürnberg

	Stimme gar nicht zu 1	Stimme weniger zu 2	Stimme teilweise zu 3	Stimme weitest-gehend zu 4	Stimme voll zu 5	kann ich nicht beurteilen
**Nachhaltigkeit**						
55. In unserer Kommune gibt es mindestens eine Person, die für das Projekt verantwortlich ist. *(Beispiel: Koordinierungsstelle, Kümmerin)*	☐	☐	☐	☐	☐	☐
56. Die Koordinierungsstelle ist dauerhaft in der Kommune verankert.	☐	☐	☐	☐	☐	☐
57. Ein weiteres Jahr ist finanziell gesichert.	☐	☐	☐	☐	☐	☐

BIG Spezifische Fragen

Nur von Kommunen auszufüllen, die am **BIG-Projekt** teilnehmen

Bitte geben Sie an, inwieweit die folgenden Kriterien **in den letzten 12 Monaten** in Ihrem Projekt erfüllt wurden

	Stimme gar nicht zu 1	Stimme weniger zu 2	Stimme teilweise zu 3	Stimme weitest-gehend zu 4	Stimme voll zu 5	kann ich nicht beurteilen
58. Das Ziel von BIG in unserer Kommune ist es, Frauen in Bewegung zu bringen.	☐	☐	☐	☐	☐	☐
59. Unsere Angebote haben bei Bedarf eine Kinderbetreuung.	☐	☐	☐	☐	☐	☐
60. Über unsere BIG-Aktivitäten wird in verschiedenen Sprachen informiert.	☐	☐	☐	☐	☐	☐
61. Im vergangenen Jahr haben sich Frauen zur "Sportassistentin interkulturell", Übungsleiterin und / oder Rettungsschwimmerin ausbilden lassen.	☐ Ja			☐ Nein		

Herzlichen Dank für Ihre Teilnahme!

© 2022 / Department für Sportwissenschaft und Sport, Friedrich-Alexander-Universität Erlangen-Nürnberg
