# Supplementary material for: Scaling Up Physical Activity Promotion Projects on the Community Level for Women in Difficult Life Situations and Older People: BIG-5 and GET-10—A Study Protocol
Source: Front Public Health. 2022 Apr 14;10:837982. doi: 10.3389/fpubh.2022.837982 (PMC9046678; doi:10.3389/fpubh.2022.837982)
Supplement: Supplementary file 4 [file Data_Sheet_3.PDF]

Friedrich-Alexander-Universität
Erlangen-Nürnberg

DSS
Department für
Sportwissenschaft
und Sport

Capacity Building in the community setting

Aim of this survey is the measurement of structures and capacities of communities for the implementation of preventive health care measures and health promotion projects.

Data will mainly function as a documentation of the status-quo and the developments of communities that take part in the BIG-5 and GET-10 projects.

The self-assessment comprises the following topics:

- **Participation** (main elements, personal initiative, participation of target population)
- **Local leadership** (Aims, content-related and organizational processes, control-competence)
- **Available resources** (environment, resources),
- **Networking and cooperation** (local and supralocal networking and cooperation, public relations),
- **Health care** (Provision of courses, Overcoming barriers, Sustainability)

The questions will ask you about your community's **situation within the last 12 months.** Think about which answer fits your situation best and check the corresponding box.

We are aware that the results represent your subjective estimation.

If you feel like you cannot answer a question, please tick "cannot answer"

Thank you for your participation!

© 2022 / Department für Sportwissenschaft und Sport, Friedrich-Alexander-Universität Erlangen-Nürnberg

PARTICIPATION

Please indicate to what extent the following criteria apply to either your BIG or GESTALT project **during the last 12 months.**

	Strongly disagree 1	Disagree 2	Neither agree nor disagree 3	Agree 4	Strongly agree 5	Cannot answer
**Main elements of the project**						
1. The project creates and strengthens structures for long-term health promotion in our community.	☐	☐	☐	☐	☐	☐
2. The needs of the target population are in focus of all project activities.	☐	☐	☐	☐	☐	☐
3. Sufficient planning sessions are taking place during which courses are being planned	☐	☐	☐	☐	☐	☐
4. The moderation contributes to the fact that all participants equally contribute to the planning meeting.	☐	☐	☐	☐	☐	☐
5. The participation of the target group in the planning and implementation of the project is being promoted by effective activation techniques.	☐	☐	☐	☐	☐	☐
6. Engagement and contribution are recognized and rewarded in the implementation of the project	☐	☐	☐	☐	☐	☐
7. People from the responsible offices and institutions are committed to the project.	☐	☐	☐	☐	☐	☐
8. There is a network of multipliers who promote the project to the target population.	☐	☐	☐	☐	☐	☐

© 2022 / Department für Sportwissenschaft und Sport, Friedrich-Alexander-Universität Erlangen-Nürnberg

	Strongly disagree 1	Disagree 2	Neither agree nor disagree 3	Agree 4	Strongly agree 5	Cannot answer
**Personal Initiative**						
9. As the coordinator, I take the initiative to solve the problems I perceive in the setting.	☐	☐	☐	☐	☐	☐
10. I am taking on more and more responsibility for the project.	☐	☐	☐	☐	☐	☐
11. As a coordinator, I contribute my needs and concerns in the planning meetings.	☐	☐	☐	☐	☐	☐
**Participation of target population**						
12. The target group is actively involved in the planning and implementation of the offers.	☐	☐	☐	☐	☐	☐

LOCAL LEADERSHIP

Please indicate to what extent the following criteria apply to either your BIG or GESTALT project **during the last 12 months.**

	Strongly disagree 1	Disagree 2	Neither agree nor disagree 3	Agree 4	Strongly agree 5	Cannot answer
**Content-related and organizational processes**						
13. I use the provided manual to implement the project.	☐	☐	☐	☐	☐	☐
14. The planning group is the central method for implementing the project's goals.	☐	☐	☐	☐	☐	☐
15. Overarching goals were defined in the planning session	☐	☐	☐	☐	☐	☐
16. Concrete goals and measures are defined in the planning group.	☐	☐	☐	☐	☐	☐
17. The planning group agreed on a concrete timeline of goals achievement	☐	☐	☐	☐	☐	☐
18. During planning meetings it is clarified who is responsible for implementing a measure.	☐	☐	☐	☐	☐	☐
**Control-competence of local leadership**						
19. The project is located at the right organization within the setting.	☐	☐	☐	☐	☐	☐
20. The local coordination manages to advance change processes in the setting.	☐	☐	☐	☐	☐	☐
21. The content of planning sessions is being logged	☐	☐	☐	☐	☐	☐
22. There is an annual report on project's activities	☐	☐	☐	☐	☐	☐
23. Defined goals and applied methods are reflected periodically.	☐	☐	☐	☐	☐	☐
24. The contents and results of the planning group are passed on and communicated within the municipality.	☐	☐	☐	☐	☐	☐
25. The achievement of goals is checked periodically.	☐	☐	☐	☐	☐	☐

© 2022 / Department für Sportwissenschaft und Sport, Friedrich-Alexander-Universität Erlangen-Nürnberg

AVAILABLE RESOURCES

Please indicate to what extent the following criteria apply to either your BIG or GESTALT project **during the last 12 months.**

	Strongly disagree 1	Disagree 2	Neither agree nor disagree 3	Agree 4	Strongly agree 5	Cannot answer
**Environmental factors**						
26. There is enough information on health and social inequalities of our community.	☐	☐	☐	☐	☐	☐
27. There is a need for health-promoting measures for the target population in our community.	☐	☐	☐	☐	☐	☐
28. There is strong political support for the project in our community.	☐	☐	☐	☐	☐	☐
**Resources**						
29. There is sufficient space to implement the exercise measures.	☐	☐	☐	☐	☐	☐
30. There is sufficient equipment for the implementation of exercise classes in the community.	☐	☐	☐	☐	☐	☐
31. There is sufficient staff for the coordination of the project.	☐	☐	☐	☐	☐	☐
32. There is sufficient financial support for the implementation of exercise classes.	☐	☐	☐	☐	☐	☐
33. There are sufficient trainers for the exercise classes.	☐	☐	☐	☐	☐	☐
34. The cost of the project is in good relation to the results that are achieved by the project.	☐	☐	☐	☐	☐	☐

© 2022 / Department für Sportwissenschaft und Sport, Friedrich-Alexander-Universität Erlangen-Nürnberg

NETWORKING AND COOPERATION

Please indicate to what extent the following criteria apply to either your BIG or GESTALT project **during the last 12 months.**

	Strongly disagree 1	Disagree 2	Neither agree nor disagree 3	Agree 4	Strongly agree 5	Cannot answer
**Local Networking and Cooperation**						
35. All participants are open to exchange.	☐	☐	☐	☐	☐	☐
36. The people and/or institutions involved form alliances and partnerships within the community.	☐	☐	☐	☐	☐	☐
37. Networking and cooperation between local people and/or institutions is excellent.	☐	☐	☐	☐	☐	☐
38. The local partners have the necessary cooperation skills.	☐	☐	☐	☐	☐	☐
39. The local partners use existing networks to overcome problems and discover new resources for the project.	☐	☐	☐	☐	☐	☐
40. The local partners work together effectively and goal oriented.	☐	☐	☐	☐	☐	☐
**Supralocal networking and cooperation**						
41. There is a national exchange with project coordinators from other communities	☐	☐	☐	☐	☐	☐
**Public Relations**						
42. I am satisfied with the public perception of the project in our community.	☐	☐	☐	☐	☐	☐

© 2022 / Department für Sportwissenschaft und Sport, Friedrich-Alexander-Universität Erlangen-Nürnberg

HEALTH CARE

Please indicate to what extent the following criteria apply to either your BIG or GESTALT project **during the last 12 months.**

	Strongly disagree 1	Disagree 2	Neither agree nor disagree 3	Agree 4	Strongly agree 5	Cannot answer
**Provision of courses**						
43. In our community there are several offers to promote the health of the target group.	☐	☐	☐	☐	☐	☐
44. The classes in our community are well attended.	☐	☐	☐	☐	☐	☐
45. Once a class has started it remains as an offer.	☐	☐	☐	☐	☐	☐
46. People who attend a class once stick with it.	☐	☐	☐	☐	☐	☐
47. The project reaches disadvantaged target groups.	☐	☐	☐	☐	☐	☐
48. Anyone in the target group who is interested can participate.	☐	☐	☐	☐	☐	☐
**Overcoming barriers**						
49. Our offers are inexpensive.	☐	☐	☐	☐	☐	☐
50. Our offers are close to the home of participants	☐	☐	☐	☐	☐	☐
51 Our offers can be attended without a contract.	☐	☐	☐	☐	☐	☐
52. Our offers can be paid in cash.	☐	☐	☐	☐	☐	☐
53. Our offers correspond to the wishes of the participants.	☐	☐	☐	☐	☐	☐
54. The multipliers and peers promote the project in different areas of life.	☐	☐	☐	☐	☐	☐

© 2022 / Department für Sportwissenschaft und Sport, Friedrich-Alexander-Universität Erlangen-Nürnberg

	Strongly disagree 1	Disagree 2	Neither agree nor disagree 3	Agree 4	Strongly agree 5	Cannot answer
**Sustainability**						
55. In our community there is at least one person who is responsible for the project.	☐	☐	☐	☐	☐	☐
56. The coordination is permanently anchored in the community.	☐	☐	☐	☐	☐	☐
57. Another year is financially secured.	☐	☐	☐	☐	☐	☐

BIG Specific Questions

PLEASE ONLY ANSWER IF YOUR COMMUNITY IS IMPLEMENTING THE BIG-PROJECT

Please indicate to what extent the following criteria apply to either your BIG or GESTALT project **during the last 12 months.**

	Strongly disagree 1	Disagree 2	Neither agree nor disagree 3	Agree 4	Strongly agree 5	Cannot answer
58. The goal of BIG in our community is to to promote women to get physically active	☐	☐	☐	☐	☐	☐
59. If required our classes have additional childcare.	☐	☐	☐	☐	☐	☐
60. Information about our BIG activities is provided in various languages.	☐	☐	☐	☐	☐	☐
61. In the past year, women have been trained as “intercultural sports assistants”, trainers and/or lifeguards.	☐ Yes			☐ No		

Thank you for taking part!

© 2022 / Department für Sportwissenschaft und Sport, Friedrich-Alexander-Universität Erlangen-Nürnberg
